# DISCO App: study protocol for a randomized controlled trial to test the effectiveness of a patient intervention to reduce the financial burden of cancer in a diverse patient population

**DOI:** 10.1186/s13063-021-05593-y

**Published:** 2021-09-17

**Authors:** Lauren M. Hamel, David W. Dougherty, Seongho Kim, Elisabeth I. Heath, Lorna Mabunda, Eyouab Tadesse, RaeAnn Hill, Susan Eggly

**Affiliations:** 1 Wayne State University School of Medicine/Karmanos Cancer Institute, 4100 John R St., Detroit, MI 48201 USA; 2 Dana-Farber Cancer Institute, Boston, MA USA; 3 Wayne State University School of Medicine, Detroit, MI USA

**Keywords:** Financial toxicity, Patient active participation, Question prompt list, Cancer treatment cost discussion, App-based intervention, Randomized controlled trial

## Abstract

**Background:**

Financial toxicity, the material and psychological burden of the cost of treatment, affects 30–50% of people with cancer, even those with health insurance. The burden of treatment cost can affect treatment adherence and, ultimately, mortality. Financial toxicity is a health equity issue, disproportionately affecting patients who are racial/ethnic minorities, have lower incomes, and are < 65 years old. Patient education about treatment cost and patient-oncologist cost discussions are recommended as ways to address financial toxicity; however, research shows cost discussions occur infrequently (Altice et al. J Natl Cancer Inst 109:djw205, 2017; Schnipper et al. J Clin Oncol 34:2925-34, 2016; Zafar et al. Oncologist 18:381-90, 2013; American Cancer Society Cancer Action Network 2010). Our overall goal is to address the burden of financial toxicity and work toward health equity through a tailorable education and communication intervention, the DISCO App. The aim of this longitudinal randomized controlled trial is to test the effectiveness of the DISCO App on the outcomes in a population of economically and racially/ethnically diverse cancer patients from all age groups.

**Methods:**

Patients diagnosed with breast, lung, colorectal, or prostate cancer at a NCI-designated comprehensive cancer center in Detroit, MI, will be randomized to one of three study arms: one usual care arm (arm 1) and two intervention arms (arms 2 and 3). All intervention patients (arms 2 and 3) will receive the DISCO App before the second interaction with their oncologist, and patients in arm 3 will receive an intervention booster. The DISCO App, presented on an iPad, includes an educational video about treatment costs, ways to manage them, and the importance of discussing them with oncologists. Patients enter socio-demographic information (e.g., employment, insurance status) and indicate their financial concerns. They then receive a tailored list of questions to consider asking their oncologist. All patients will have up to two interactions with their oncologist video recorded and complete measures at baseline, after the recorded interactions and at 1, 3, 6, and 12 months after the second interaction. Outcome measures will assess discussions of cost, communication quality, knowledge of treatment costs, self-efficacy for treatment cost management, referrals for support, short- and longer-term financial toxicity, and treatment adherence.

**Discussion:**

If effective, this intervention will improve awareness of and discussions of treatment cost and alleviate the burden of financial toxicity. It may be especially helpful to groups disproportionately affected by financial toxicity, helping to achieve health equity.

**Trial registration:**

ClinicalTrials.gov NCT04766190. Registered on February 23, 2021

## Background

Financial toxicity, the severe material and psychological burden of the cost of cancer treatment, affects an estimated 30–50% of patients [1–5]. As cancer treatment costs escalate [6] and the cost burden increasingly shifts to the patient [7–10], more patients are experiencing severe material economic consequences. Across cancer types, patients are, on average, responsible for $16,000 annually for out-of-pocket direct and indirect treatment costs [11]. People with cancer are 2.6 times as likely to file for bankruptcy as people without cancer [12, 13]. Recent studies of breast cancer survivors found that 24% used all of their savings over a 6-month period to pay for the treatment [14], and 62% of colorectal cancer survivors incurred debt to pay for the treatment with an average liability of $26,860 [15]. Financial toxicity can also result from indirect costs, such as loss of income. Breast cancer survivors reported losing an average of 42 work days per year, which translated to an average of $8236 in lost wages [16]. Treatment costs can also have deleterious psychological effects, with almost half of survivors reporting significant, even catastrophic, levels of cost-related distress [17–19]. We emphasize that the material and psychological consequences of financial toxicity can be experienced both short term during diagnosis and treatment and longer term into survivorship [1, 19, 20].

The burden of financial toxicity is a health equity issue, disproportionately affecting patients who are racial/ethnic minorities [15, 21–24], have lower incomes [13, 15, 18, 22], and/or are < 65 years of age [13, 22, 25]. Compared to White cancer patients, Black cancer patients are twice as likely to deviate from treatment, have utilities turned off, and move out of their homes because they cannot afford to pay for the treatment and living expenses [21]. Black survivors are more likely to report treatment-related debt (15%) than White survivors (9%). Lower-income Black breast cancer patients spend a greater proportion of their income (27–31%) on treatment expenses than lower-income Whites (9–13%) [22]. Survivors are 1.4 times as likely to be unemployed (often due to extended time off for treatment/recovery) as people without cancer; racial/ethnic minority survivors are twice as likely to be unemployed as White cancer survivors [26]. The disproportionate burden of financial toxicity experienced by racial/ethnic minorities remains even when controlling for employment status and insurance status at diagnosis [22, 23]. Younger patients (< 65) are also at greater risk for financial toxicity and bankruptcy than older patients, mainly due to insurance status (i.e., Medicare) [25]. A study of colon cancer patients found being younger, non-White, and/or having a low annual income increased the risk of financial toxicity [15]. A study of breast cancer survivors found the burden of financial toxicity was higher for younger patients with lower incomes [27].

Cancer treatment costs and related material and psychological burden influence treatment recommendations [28], treatment decisions [29–32], adherence [1, 3, 20, 32], and mortality [25]. A majority of oncologists report that cancer drug costs (56%) and patient out-of-pocket costs (84%) influence their treatment recommendations [28]. Costs also influence patients’ treatment decisions [29–32], including whether to participate in clinical trials [30, 33]. Patients with lower incomes are more likely to choose treatments with lower costs even if those treatments have lower survival and higher toxicity [31]. To offset cost, patients may deviate from treatment (including treatment for side effects) [3, 34, 35] and/or forgo treatment altogether [32]. A study of 254 patients being treated with either chemotherapy or hormonal therapy found that 20% of patients took less than the prescribed amount of medication, partially filled, or avoided filling prescriptions due to the out-of-pocket costs [3]. Another study of patients being treated for solid tumors found that 45% of patients were non-adherent to treatment due to cost [20]. A study of 1556 cancer survivors found that those who reported financial problems were more likely to delay (18.3% vs. 7.4%) or forgo treatment (13.8% vs. 5.0%) compared to respondents without financial problems [36]. In a study of more than 22,000 women with early-stage breast cancer, higher copayments were associated with greater non-adherence to treatment by Medicare and non-Medicare patients. Indirect costs (e.g., travel distance) also reduce the likelihood of receiving or completing treatment [37]. Severe financial distress resulting from cancer treatment may itself be a mortality risk factor [25].

Health insurance, whether public or private, does not protect patients against financial toxicity [1, 4]. The American Cancer Society conducted a national poll of 1000+ adults who reported they or a member of their household had cancer or a history of cancer [4]. Regardless of insurance, 20% of respondents had difficulty paying for basic necessities, 15% used up all or most of their savings, and 11% incurred thousands of dollars of debt due to treatment expenses. This survey found that 26% of respondents who were insured during their cancer diagnosis and treatment experienced problems with their coverage [4]. A study of 10,000 patients with Medicare or private insurance found higher copayments were related to prematurely stopping oral chemotherapy [38].

Including costs as part of patient-oncologist treatment discussions could help raise awareness and prepare patients to manage treatment costs. A major contributor to the burden of financial toxicity is patients’ lack of awareness of potential costs they may incur during treatment and survivorship and how to manage those costs [2, 39–42]. Patients are often unprepared when out-of-pocket costs arise [43]. Patient-oncologist treatment cost discussions could improve patients’ knowledge of what costs to anticipate [2, 39, 41, 42, 44] and connect patients with vital financial resources [43]. Most patients express a desire to discuss cost with their physicians [45–47]. However, a rich body of research, including our own, shows that cost discussions occur infrequently [48–50]. In a study of video-recorded treatment discussions (*n* = 103), we (Hamel et al. [48]) found that cost discussions occurred in only 45% of treatment discussions. When cost was discussed, it was mostly patient-initiated (63%) and focused more on indirect costs (e.g., time off work) than on direct costs (e.g., copayments) [48].

In an attempt to increase patient awareness and communication about cost, the American Society of Clinical Oncology (ASCO) developed tools, including ASCO Answers: Managing the Cost of Cancer Care, [51] ASCO’s Value Framework, [2] and ASCO’s Patient-Clinician Communication Consensus Guidelines [52]. These materials are intended to educate patients on the types of treatment costs they may incur, to encourage physicians to discuss patient cost concerns directly, and to refer patients to a social worker or financial navigator, if needed. Unfortunately, ASCO’s current materials are static, text-heavy, and do not provide patients with specific actions they can take to manage cost. Though they encourage discussions, the guidelines are overly general and do not provide patients and physicians with specific strategies to initiate such discussions.

Increasing patient active participation during oncology interactions has the potential to improve the frequency and quality of patient-oncologist treatment cost discussions [53, 54]. Research on clinical interactions in many medical settings shows patient active participation (e.g., asking questions, expressing concerns, making assertions) plays an important role in short-, intermediate-, and long-term outcomes [55, 56]. Patient active participation influences the amount of information physicians provide [57–59], the treatment physicians recommend [60], topics patients and physicians discuss [54, 61, 62], patient healthcare decisions [63], and patient psychosocial and physical health outcomes [64, 65]. In this proposal, we build on prior research on patient active participation by providing education along with prompting for active participation in clinical interactions. Research shows that, in the short term, cost education and patient-oncologist discussions can improve patient self-efficacy for managing cost [44, 66], increase referrals for support (e.g., social work) [43], and reduce cost distress and perceived material hardship [67]. Longer-term effects include improved financial toxicity [67] and treatment adherence [68].

There is a great need for tools to improve patient treatment cost education and to prompt patient-oncologist treatment cost discussions. Question prompt lists (QPLs) are communication tools designed to enhance patient active participation in interactions with physicians. QPLs are lists of questions that patients might consider asking their healthcare provider during a clinical interaction [62, 69–72] and have been shown to improve patient active participation in interactions [53], psychological outcomes (e.g., anxiety) [61], cognitive outcomes (e.g., information recall) [69], patients’ report of their role in treatment decisions [70], and trust in their oncologist [69, 71, 72]. Our own research demonstrated the success of QPLs in increasing patient active participation among Black patients with cancer discussing treatment with oncologists [53]. We improve upon the effectiveness of currently published QPLs in three ways: (1) we include education on issues of costs, so patients are aware of the need to ask questions about cost to gain information and support; (2) we specifically address treatment costs in the list of questions; and (3) we use an electronic format that is scalable and can be tailored to a specific patient’s needs. We propose to address the limitations of current ASCO tools by increasing the knowledge of costs and discussions through an intervention comprising an “app”-based educational video and QPL focused on treatment cost. This tailorable [69] app, DIScussions of COst App (DISCO App) [73], was developed in collaboration with survivors, clinicians, and a software development firm and pilot-tested in two outpatient oncology clinics. Based on our community-engaged development process and pilot tests, we believe this app-based intervention may be particularly effective in a diverse patient population. Smartphone/tablet technology use is highly prevalent across racial/ethnic groups, ages, and income levels [74–78], as is willingness to use these technologies for health interventions [79, 80]. Interventions tailored to an individual are also more effective at prompting behavior change in diverse populations, compared to static interventions [81–83].

### Conceptual model

**Fig. 1:**
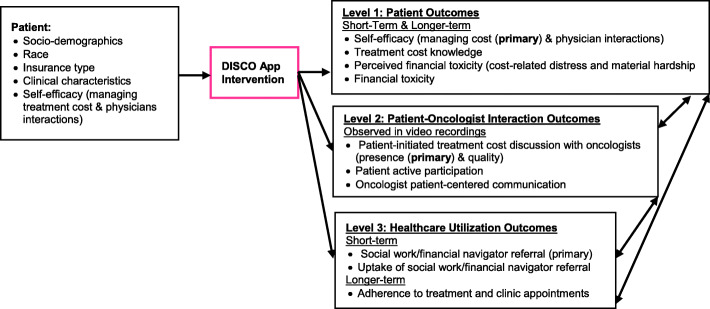
Conceptual model

Financial toxicity comprised psychological and material economic burden, both of which can contribute to poor treatment adherence and mortality. Our overall goal is to address the burden of financial toxicity and work toward health equity through a tailorable education and communication intervention. Our conceptual model (Fig. 1) illustrates our expectation that patient baseline characteristics (e.g., socio-demographics, race, insurance type, clinical characteristics, self-efficacy in managing treatment cost and physician interactions, treatment cost knowledge) and the DISCO App intervention, provided just prior to the second patient-oncologist interaction, will improve short- and longer-term outcomes at three levels. At the patient level, short- and longer-term outcomes include self-efficacy for managing cost and physician interactions, treatment cost knowledge, perceived financial toxicity [cost distress, material hardship], and actual financial toxicity. At the patient-physician interaction-level outcomes include patient active participation, frequency and quality of patient-initiated cost discussions with the oncologist, and oncologists’ patient-centered communication. At the healthcare utilization level, short-term outcomes include social worker/financial navigation (SW/FN) referrals and SW/FN referral uptake. Longer-term outcomes include adherence to treatment and to clinic appointments.

### Study overview

**Fig. 2:**
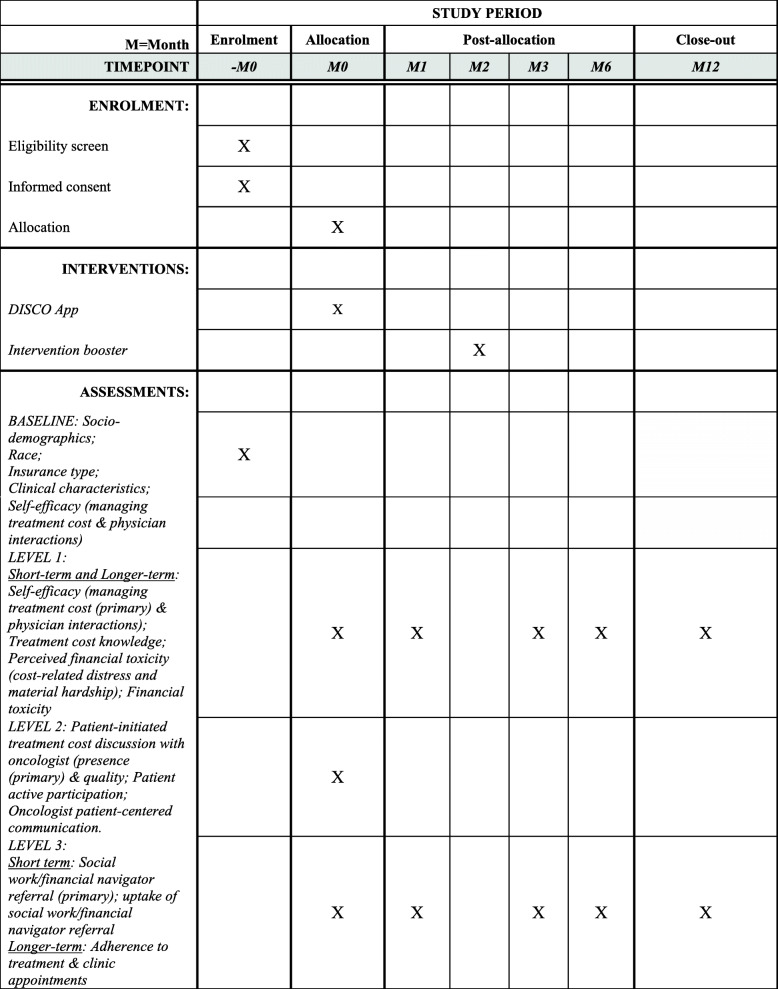
SPIRIT figure for the study period for patient participants

**Fig. 3:**
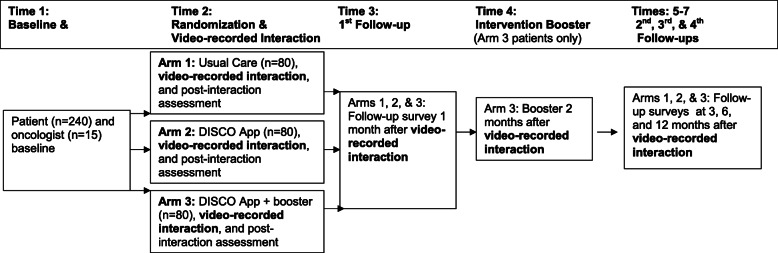
Flow diagram of participant enrollment, randomization, and procedures

**Fig. 4:**
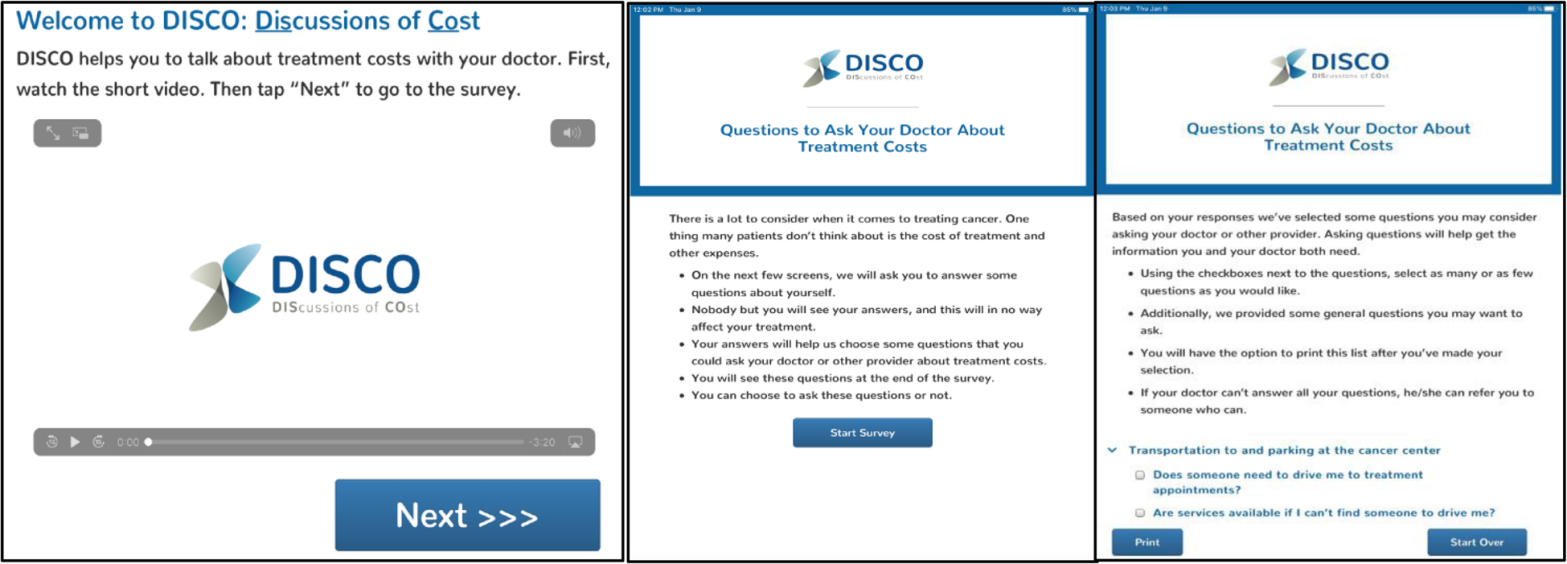
DISCO App educational video and QPL introduction screens

The DISCO App [73] is designed to improve patient awareness of potential treatment costs and patient-oncologist treatment cost discussions and, in turn, other outcomes. The current study will test the DISCO App’s effectiveness on short- and longer-term patient outcomes (with and without the addition of an intervention booster) through a longitudinal randomized controlled trial (RCT) (Figs. 2, 3, and 4). More specifically, the study is designed to achieve the following aims and test the following hypotheses:

1. Aim 1: Determine the effectiveness of the Discussions of Cost App (DISCO App) on short-term outcomes at three levels:
  - Patient (treatment cost knowledge, self-efficacy for managing cost and physician interactions, and perceived financial toxicity [cost distress, material hardship])
  - Patient-oncologist interaction (patient active participation, frequency and quality of patient-initiated cost discussions with oncologists, oncologists’ patient-centered communication as observed in video recordings)
  - Healthcare utilization (social work/financial navigation referrals, social work/financial navigation referral uptake)
  We hypothesize that:

- H1a: The DISCO App will significantly improve the outcomes for all intervention patients as compared to usual care patients.
- H1b: The DISCO App will significantly improve the outcomes for patient groups suffering the disproportionate burden of disparities in the financial consequences of cancer care, specifically:
  - H1b1: Black patients will experience significantly greater improvement than White patients.
  - H1b2: Lower-income patients will experience significantly greater improvement than higher-income patients.
  - H1b3: Younger patients will experience significantly greater improvement than older patients.

2. Aim 2: Determine the effectiveness of the DISCO App on longer-term outcomes (financial toxicity, treatment adherence, and clinic appointment adherence). We will compare longer-term outcomes across arms.

We hypothesize that:

- H2a: Patients receiving the DISCO App + booster will experience the greatest improvement in the outcomes, followed by patients receiving the DISCO App, and last, usual care patients.
- H2b: Patient groups suffering the disproportionate burden of disparities in the financial consequences of cancer care will experience the greatest improvement in outcomes, specifically:
- H2b1: Black patients will experience significantly greater improvement than White patients.
- H2b2: Lower-income patients will experience significantly greater improvement than higher-income patients.
- H2b3: Younger patients will experience significantly greater improvement than older patients.

3. Aim 3: Test potential mediators and moderators of the relationship between the short-term and longer-term outcomes of the DISCO App.
  - H3: We hypothesize that the DISCO App will increase the frequency and quality of patient-initiated cost discussions, which will increase social work/financial navigation referrals, social work/financial navigation referral uptake, and patient self-efficacy for managing treatment cost, which in turn will reduce financial toxicity and improve adherence.
  - H4: We hypothesize that this relationship will be moderated by patient socio-demographic characteristics.

## Methods and design

### Study design

This is a clinical trial involving a behavioral intervention focused on patients (with and without an intervention booster), which will be evaluated with a longitudinal between-subjects randomized controlled trial in which patients will be randomized to intervention or usual care groups, and outcomes are compared between the groups. All study procedures have been approved by Wayne State University’s (WSU) IRB (IRB-20-2836). SPIRIT guidelines were used to report this protocol [84].

### Participants and setting

This trial will be conducted at WSU/Karmanos Cancer Institute (KCI), a NCI-designated comprehensive cancer center located in Detroit, MI, USA, which serves a highly diverse population.

#### Medical oncologist eligibility criteria

We will recruit up to 15 medical oncologists and medical oncology fellows at the beginning of data collection, prior to patient recruitment. Medical oncologists are eligible to participate if they treat patients with breast, prostate, lung, or colorectal cancers at KCI. We focus on these cancers because they are the leading sites of cancer cases and deaths in the USA and because, as solid-tumor cancers, their treatment protocols are similar [85]. Medical oncologists who do not treat patients with these cancers at KCI will not be eligible to participate.

**Fig. 5:**
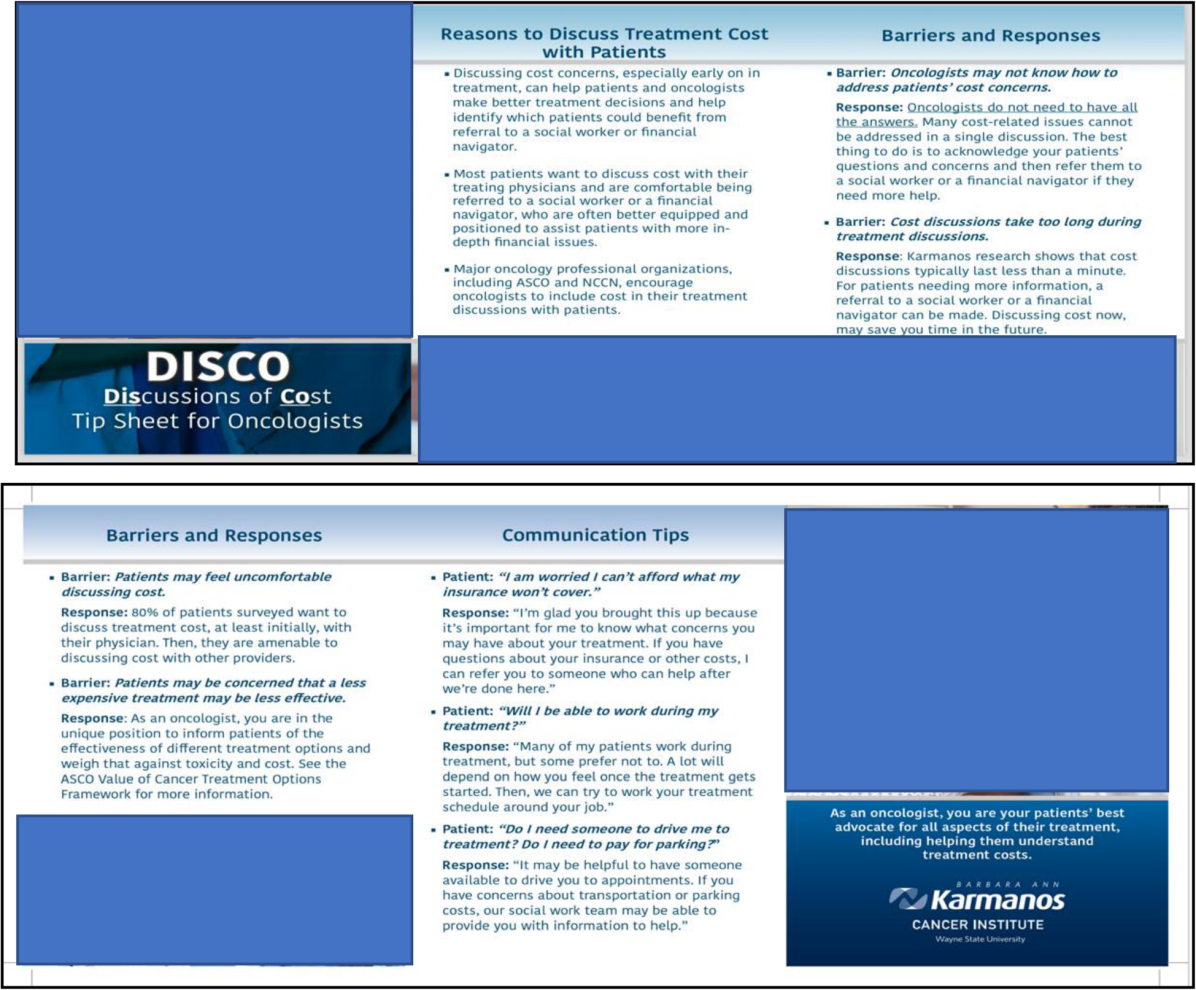
Oncologist tip

To recruit oncologists, research staff will explain the study at clinic program meetings and meet with interested oncologists individually to answer questions and obtain consent. Oncologists who consent will agree to (1) complete a baseline survey, (2) inform (or designate a clinical member of the study team to inform) their eligible new patients (via a phone call, e-mail, or face-to-face conversation) about the study prior to their initial appointment to discuss treatment, (3) have interactions with participating patients video recorded, and (4) complete a brief survey following interactions with participating patients. Upon recruitment, oncologists will receive a “tip sheet” to help prepare them for treatment cost discussions with patients (Fig. 5). Oncologists will continue their participation throughout the study period (approximately 4 years), and they will receive a $30 gift card upon consent for their participation in the study.

#### Patient eligibility criteria

We will recruit up to 240 (120 men, 120 women) White and Black patients from various socio-economic statuses and ages. Patients are eligible if they are > 18 years of age, identify as either Black or White, are able to read and write in English, have an email account, and are newly diagnosed with breast, prostate, lung, or colorectal cancer (stages I–IV) for which systemic therapy is a likely recommended treatment. Strata will be created to ensure study arms are balanced by patient race, income, age, and sex. The majority of patients treated at KCI and living in the Detroit Metropolitan Area identify as either Black or White, and therefore, only the members of these two populations will be recruited.

Eligible patients will be identified by research staff who will review the participating oncologists’ schedules weekly. Patients will be contacted via phone call, face-to-face, or email from the participating oncologist’s clinical staff to inform them of the study and assess interest. If interested, patients will be contacted by research staff via a phone call or in person in the clinic prior to a scheduled appointment. The research staff member will explain the study and obtain consent and collect baseline data. Patient participants will be (1) asked to arrive 30 min early to their next scheduled appointment with their oncologist, (2) randomized to one of the study arms, (3) asked to complete a brief survey following the video-recorded interactions with a participating oncologist, (4) asked to have up to two of their clinical interactions video recorded, and (5) asked to complete up to four follow-up surveys at 1, 3, 6, and 12 months after their last video-recorded interaction. All patients will provide baseline socio-demographic information at the time of consent. We will ask the patients for their preferred method of contact for follow-up surveys.

Participating physicians and patients can stop their participation at any time. If a participating physician leaves the institution or stops their practice, they are no longer eligible, and if patients receive their care at another institution, they are no longer eligible.

### Procedures

Just prior to the second patient-oncologist visit to finalize treatment plans, but before treatment begins, a research assistant will use data collection software (Qualtrics) to randomize patients into one of three study arms (1:1:1). Patients in arm 1 will receive usual care, patients in arm 2 will receive the intervention, and patients in arm 3 will receive the intervention and an intervention booster. Patients in arms 2 and 3 will receive the DISCO App. Patients in arm 3 will receive an intervention booster provided 2 months after receiving the DISCO App. For all patients, we will video record the second patient-oncologist interactions using our established, unobtrusive video recording system [48, 53, 86–88].

Just before the second patient-oncologist interaction, patients in arms 2 and 3 will access the DISCO App [73] on an iPad. Our research demonstrates that patients are comfortable with iPads, especially if they are assisted [53, 89]. Research assistants will show patients the DISCO App with an explanation, instructions, and demonstration. The staff will be trained not to answer questions nor discuss cost, but to encourage patients to ask questions during their clinical interaction. These meetings will be audio-recorded to assess fidelity to the protocol. Based on the feasibility data from our pilot study, we anticipate that using the DISCO App will take < 20 min. Since patients in arms 2 and 3 may bring the printout and/or iPad into the interaction, oncologists will not be blind to the study arm. Thus, in an effort to conceal which patients are randomized to each study arm, we will give patients in arm 1 treatment information on a printout for their meeting with their oncologist. Immediately after the recorded interactions, patients and oncologists will complete brief surveys assessing any cost discussions that occurred. Patients in arms 2 and 3 will also complete assessments of the DISCO App (e.g., “The app helped me ask my treatment cost questions”) after the second interaction.

All patients will be contacted via their preferred method of contact to complete follow-up measures including questions about their disease and treatment status, whether they received a referral for SW/FN; if they followed up on that referral, self-efficacy managing treatment cost; and perceived short-term financial toxicity (perceived material hardship and cost distress) in the first follow-up, actual financial toxicity in the remaining follow-ups, and treatment adherence. Follow-up will occur at 1, 3, 6, and 12 months after the second recorded interaction. Patients who receive the intervention booster will also complete booster assessments (e.g., “The email reminded me of helpful information”).

### Intervention

#### The DISCO App

The DISCO App (Fig. 4) is displayed on an iPad provided to intervention patients in a private room just prior to their second interaction with their oncologist to discuss and finalize the treatment plans. The DISCO App opens with an introduction screen. First, patients watch a 3-min educational video featuring a communication scientist, medical oncologist, and a patient using the DISCO App. The video summarizes the types of treatment costs patients may incur (e.g., copayments, transportation/parking costs, time away from work) and ways to manage those costs (e.g., talk with an oncologist or social worker, contact pharmaceutical companies, seek clarification from insurance provider). The video ends by emphasizing to patients that the best way to start managing treatment costs is to discuss them with their oncologist who can answer their questions or refer them to someone who can assist. Second, after the video, the QPL is introduced with the following text: “There is a lot to consider when it comes to treating cancer. One thing many patients don’t think about is the cost of treatment and other expenses.” The text continues to explain that the DISCO App includes a short survey, which will lead to some cost-related questions the patient can consider asking the oncologist. This section asks patients to enter their demographic information and their financial characteristics. Specifically, patients respond to 17 questions (e.g., “How much do you know about your insurance coverage?”; “Are you currently employed?”; “Is there anyone who helps you when you’re sick or need help of any kind?”). Based on patient responses, an individually tailored QPL with up to 18 cost-related questions in 7 categories is generated (Table 1). For example, patients who indicate they are employed will be prompted to ask: “Can I schedule my treatment around my job?”; patients who indicate transportation concerns will be prompted to ask: “are services available if I can’t find someone to drive me?”; patients who indicate they are unfamiliar with their insurance coverage will be prompted to ask: “Is there someone I can talk to about my insurance and treatment cost questions?” All patients will be provided with four diagnosis questions (e.g., “What is my diagnosis?”), have the option of adding in any of their own questions, and then either take the iPad or a printed question list into the meeting with the oncologist. Thus, the DISCO App arms patients with concrete information about the types of out-of-pocket and indirect costs they may incur while undergoing treatment, specific actions they can take to begin to address those costs, and a list of individually tailored cost-focused questions they can take with them to the clinic visit to ask their oncologist. This information and individualized prompting are something few patients with cancer currently receive, on any topic.

**Table 1:** The DISCO App’s prompted questions by question type

**Cost of appointments and treatments**	
1. How much will I have to pay for my treatment?	
2. Is there a less expensive drug, like a generic, that will be equally effective?	
3. How many visits will I have? I may have to pay each time I come to the cancer center (co-pay, parking, etc.).	
4. What happens if I can’t pay for some of my treatment costs?	
**Help with understanding my treatment costs and what my insurance covers**	
5. Do I need additional or supplemental insurance coverage?	
6. Do I have a co-pay every time I come to the cancer center?	
7. Is there someone I can talk to about my questions about my insurance and treatment costs?	
**Transportation to and parking at the cancer center**	
8. Does someone need to drive me to treatment appointments?	
9. Are services available if I can’t find someone to drive me?	
10. How much does parking cost?	
**Living far from the cancer center**	
11. Is it possible for me to receive my treatment closer to where I live?	
12. Are there free or reduced-cost hotels nearby for me and my family?	
**Working during treatment**	
13. Can I keep working during treatment? If not, when can I go back to work?	
14. Can I schedule my treatment around my job?	
15. Do I need to file Family and Medical Leave Act (FMLA) paperwork? If so, how?	
**Assistance programs**	
16. Are assistance programs available to help me with treatment costs or other expenses or needs?	
17. If I need a wig or other supplies, is there somewhere I can get them free or at a reduced cost?	
**Family and living responsibilities**	
18. Can I schedule my treatment around my family’s schedule?	
**General questions about cancer and treatment (all patients will get these)**	
19. What is my diagnosis and stage?	
20. Is it possible to cure my cancer?	
21. What is my treatment plan?	
22. Are there clinical trials I can participate in? If so, will this cost more or less than standard treatment?	

Financial toxicity is multifaceted and long-term. We expect the DISCO App to influence short-term outcomes, but we expect it may need reinforcement to influence longer-term outcomes (e.g., financial toxicity, treatment adherence). Thus, we will explore the effects of a booster to reinforce the effects of the DISCO App. Patients in arm 3 will receive the booster 2 months after receiving the DISCO App. The booster will be a tailored email or text message reminding patients of (1) the content in the educational video, (2) the questions they selected, and (3) that treatment costs are something they can discuss with their oncologist.

#### Oncologist tip sheet

During DISCO App [73] acceptability testing, some participants thought the DISCO App would be useful for patients to prompt treatment cost discussions with their oncologist and gain important information for their treatment. However, they expressed the concern that oncologists may be unprepared to answer cost questions. In response, we designed an oncologist “tip sheet,” which emphasizes oncologists’ role in cost discussions (as recommended by ASCO) and provides ways to overcome identified barriers to cost discussions (Fig. 5) [90, 91]. For example, oncologists report concern that they will be unable to answer questions about treatment costs. It is impractical to expect oncologists to know the complexities of treatment cost so the tip sheet provides language, including “if a patient asks about cost and you do not know the answer, you can simply say: ‘I’m glad you brought this up, because it’s important for me to know what concerns you have about your treatment. I’m not an expert in this area, but if you have questions about costs, I can arrange for you to meet with a social worker who can help after we’re done here [48].’”

We designed the tip sheet to be a two-sided, tri-fold document that fits in physicians’ white lab coats.

#### Intervention booster

Two months after receiving the DISCO App, patients in arm 3 will receive an intervention booster. The booster will be an email or text message (depending on patient preference) to remind patients (1) of the content in the education video, (2) the questions they selected from the DISCO App, and (3) that treatment costs are something they can discuss with their oncologist or other providers. The email or text message will include a “read receipt” so we can track whether the patients view the email or text message.

### Measures

Data include patient and oncologist self-report, video-recorded patient-oncologist treatment discussions, and medical chart data. Video recording allows us to use our validated coding systems [48, 53, 92] to assess the outcomes that occur during the interaction.

Most of the measures in this study have been used with cancer patients, including in the DISCO App’s feasibility pilot, with high completion rates and few complaints about burden. However, the first ten patients who complete all measures will be specifically queried about the burden. Measures will be adjusted if necessary.

#### Baseline measures

Baseline measures from patients and oncologists will be used as moderators and covariates in analyses of the intervention’s effects. Patients: After providing consent, patients will provide socio-demographics including age, race/ethnicity, gender, education, marital/personal status, income, employment, and financial situation (e.g., *It is difficult for me to live on my total household income right now*) [15]. They will also complete measures eliciting their diagnosis, their recommended treatment (if known),; insurance type, their self-efficacy in patient-physician interactions (PEPPI *α* = .91; e.g., *How confident are you in your ability to know what questions to ask your doctor?*), patient-practitioner orientation (e.g., *The doctor is the one who should decide what gets talked about during a visit*), their self-efficacy in managing the cost of treatment (adapted from a validated scale; e.g., *I am confident I can pay for the direct costs of my treatment*), [93] their level of treatment cost distress (e.g., *I am concerned about how much my cancer treatment will cost me*), and their anticipated material hardship due to their cancer treatment (e.g., *I know that I have enough money in savings, retirement, or assets to cover the costs of my treatment*) [94]. Oncologists: After providing consent, oncologists will complete a one-time assessment of their socio-demographic and professional information, including race/ethnicity, gender, age, and years in practice. Oncologists will also complete measures of patient-practitioner orientation (e.g., *The doctor is the one who should decide what gets talked about during a visit*), their perceptions of the oncologists’ role in treatment cost discussions (e.g., *Oncologists should be discussing treatment cost with their patients)*, and their self-efficacy with discussing treatment cost.

##### Medical records

We will use the patients’ medical records to abstract information on cancer diagnosis, co-morbidities, and their zip code.

#### Level 1: Patient outcome measures

Immediately after the video-recorded interactions, patients will complete the following measures: self-efficacy in patient-physician interactions; [93] self-efficacy in managing the cost of treatment; [93] knowledge of types of treatment cost (e.g., *Cancer treatment may cost me in the following ways*) and ways to manage those costs (e.g., *The following are ways I can manage treatment cost*); perceived financial toxicity, comprising treatment cost distress (5-items; e.g., *I am worried about how much my cancer treatment will cost*); and perceived material hardship (7-items; e.g., *Do you anticipate having to borrow money to pay for cancer treatment? Do you anticipate having to take unpaid time off from work for treatment?*) [15]. Intervention patients will also provide perceptions of the DISCO App after the interaction (5 items; e.g., *The DISCO App helped me ask my doctor my cost questions*) [95].

At the 1-, 3-, 6-, and 12-month follow-ups, patients will be contacted via their preferred method by research staff to complete measures on their disease and treatment status (e.g., disease status, type of treatment) to help account for any differences observed in the outcome measures. Patients will also complete measures assessing self-efficacy in patient-physician interactions, [93] self-efficacy in managing the cost of treatment, [93] their employment status, and actual financial toxicity using the 11-item COST scale (*α* = .90; e.g., *My out-of-pocket medical expenses are more than I thought they would be*) [94]. At the 3-month follow-up, patients who received the booster will be asked if they received the booster and their perceptions (e.g., *The reminder email or text message was helpful with my cost questions and concerns*).

#### Level 2: Patient-oncologist interaction outcome measures

Immediately after the video-recorded interactions, patients and oncologists will complete measures of the perceived presence of treatment cost discussion(s) (3 items; e.g., *Did you and your oncologist (patient) discuss the cost of your (his/her) cancer treatment today?*) and satisfaction with treatment cost discussion(s) (3 items; e.g., *I am satisfied with how my oncologist (patient) and I discussed treatment cost today*).

##### Observational measures

Trained research assistants (RAs), blind to research questions, will observe and rate video-recorded interactions using our established procedures to ensure acceptable inter-rater reliability [48, 53, 96–98]. To assess the frequency and quality of a cost discussion, RAs will determine if a treatment cost discussion occurred (e.g., *any verbal expression of perceived direct or indirect costs for the patient for cancer treatment*), who initiated the cost discussion (e.g., *patient, oncologist*), and what topics were discussed (e.g., *insurance, transportation, etc.*) [48]. RAs will also rate the quality of the interaction through assessing patient active participation (e.g., *the patient asked a lot of questions*) [92] and oncologists’ patient-centered communication (12 items, *α* = .75; e.g., *the doctor encouraged the patient to express concerns and worries*) [92]. Another team of trained RAs will assess if the DISCO App or printout is present and/or used [53] and interaction length [53].

#### Level 3: Health utilization outcomes

Immediately after the video-recorded interactions, patients will complete measures of whether they wanted and/or received a SW/FN referral, and if so, if they followed up on the referral.

Oncologists will complete measures on whether they made a SW/FN referral for the patient.

At the 1-, 3-, 6-, and 12-month follow-ups, patients will be contacted by their preferred method of contact by research staff to complete measures, including whether they wanted a SW/FN referral, and if so, whether they followed up on that referral; treatment adherence (Medical Outcomes Study General Adherence; e.g., *I had a hard time doing what the doctor suggested I do for treating my cancer*) and treatment cost-related adherence (e.g., *Was there a time in the past 12 months when you needed to see a doctor for your cancer but could not because of cost?*); [99] and clinical appointment adherence.

Using medical records, we will assess whether the oncologist made a SW/FN referral, and if so, if the patient followed up on the referral, treatment adherence, and clinic appointment adherence.

### Sample size calculation/analyses

**Fig. 6:**
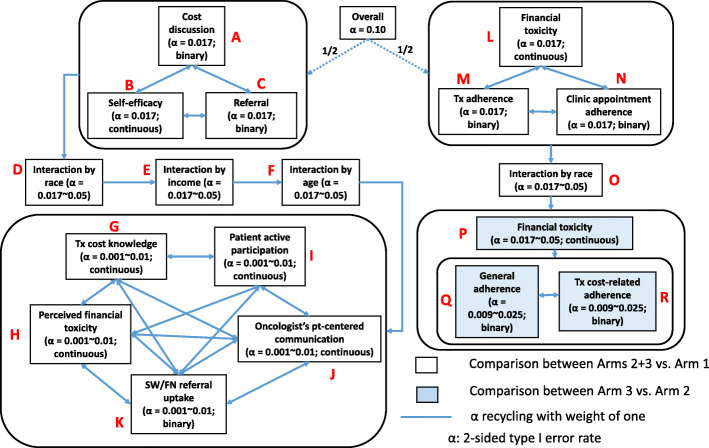
Graphical representation of Hierarchical Holm’s Testing

For the first primary objective (Fig. 6 (A–C)), we expect that the presence of a cost discussion will influence the other outcomes. Thus, the presence of cost discussions was used to estimate the sample size and power justification for other outcomes. The rates of occurrence for cost discussions and SW/FN referrals will be calculated, and the outcomes will be compared between usual care (arm 1) vs. both DISCO App and DISCO App + booster (arms 2 + 3). The sample size ratio was assumed to be 1:2 since arms 2 and 3 will be the same in the intervention evaluation. We are employing a between-subjects design with patients nested within oncologists (e.g., accounting for oncologists seeing multiple patients). The unit of analysis is the patient-oncologist interaction, and data from these interactions will likely be more similar within oncologists than between oncologists. Thus, we used multi-linear models (MLM) with a binary outcome (i.e., discussed cost or did not discuss cost) to determine the sample size justification and power analyses using an MLM cluster-randomized design for two proportions [100, 101]. The effect of intra-cluster correlation (ICC) was further examined using ICC estimation from the random intercept logistic model [102]. Based on our previous observational study [48], we assume that the ICC will be ≤ 0.04 (hereafter, for the sake of the worst scenario and the sample size estimation, we assumed that ICC is 0, which produces the largest sample size), and the rate of cost discussions without the DISCO App will be 45%. Our pilot study found that the cost discussion rate with the DISCO App is 100% (95% CI, 0.86 to 1). Based on findings from our observational and pilot studies, in this study, we expect that the rate of cost discussions will be at least 75% with the DISCO App, and the minimally meaningful difference between the two groups will be 30% [48, 103]. We consider each oncologist a “block” and assume a Bonferroni-corrected 2-sided 1.7% level (= 5%/3 primary endpoints). Thus, 180 patients (10 oncologists × 3 arms × 6 patients) will achieve at least 90% power to detect a 30% difference in the rate between the two arms. This is also what we will need to detect a difference in the primary outcome at the healthcare utilization level (SW/FN referral). A total of 180 patients will allow us to detect an effect size of ≥ 0.58 for the patient-level outcome (self-efficacy for managing treatment cost) with 90% power at a 2-sided 1.7% level. With 20% attrition, we will need 240 (10 oncologists × 3 arms × 8 patients) patients to maintain a balanced design.

For first, second, and third secondary objectives (i.e., interactions by each of race (Black [B] vs. White [W]); income (high vs. low); and age (< 65 vs. ≥ 65)) (Fig. 4(D-F)), 180 patients will produce more than 89% power to detect any of three interactions for binary outcomes when the group proportions under the alternative hypothesis are 0.45, 0.45, 0.45, and 0.75 (e.g., for arm 1-W, arm 1-B, arms 2–3-W, arms 2–3-B, respectively) using a *z*-test from a GEE analysis of a logistic model at a 2-sided 1.7% level. This will allow us to detect an interaction difference of at least 1.64 for continuous outcomes with 90% power at a 2-sided 1.7% level when an estimated SD of subjects is one.

For the fourth secondary objective (Fig. 4(G-K)), 180 patients will allow us to detect a difference of at least 0.23 between two proportions for binary outcomes as well as to detect an effect size of at least 0.72 for continuous outcomes with 90% power at a 2-sided 0.1% level.

For the second primary objective (Fig. 4(L-N)), outcomes will be measured at 5 time points (baseline, 1, 3, 6, 12 months post-intervention), and the power was justified using longitudinal logistic and continuous MLM. A total of 180 patients will have 90% power to detect a difference of 0.18 in slopes for continuous outcomes as well as to detect a difference of at least 0.13 for binary outcomes between arms 2 + 3 and arm 1 at a 2-sided 1.7% level when the autocorrelation is assumed to be 0.

For the fifth secondary objective (Fig. 4(O)), 180 patients will allow us to detect an interaction between race and slope of at least 0.52 for continuous outcomes with 90% power at a 2-sided 1.7% level. A total of 180 patients will produce more than 90% power to detect any interactions with race for binary outcomes when the group proportions under the alternative hypothesis are 0.45, 0.45, 0.45, and 0.75 (e.g., for arm 1-W, arm 1-B, arms 2–3-W, arms 2–3-B, respectively) using a normal test from a generalized estimating equations (GEE) analysis at a 2-sided 1.7% level when the autocorrelation is assumed to be 0.

For the sixth secondary objective (Fig. 4(P-R)), 180 patients will give us 90% power to detect a difference in slopes for continuous outcomes between arm 3 and arm 2 of 0.21 at a 2-sided 1.7% level. These patients will allow us to detect a difference of at least 0.16 for binary outcomes between arm 3 and arm 2 at a 2-sided 0.9% level when the autocorrelation is assumed to be 0.

All sample size and power justifications were performed by PASS 2020 (NCSS LLC, Kaysville, UT, USA). The primary analyses will be based on complete data without missing values. As a sensitivity analysis, we will also perform hypothesis testing after multiple imputation. For time-independent variables (e.g., baseline attributes), multiple imputation will be performed using chained equations (MICE) [104], and for the time-dependent variables, Amelia II will be used to consider time trends of a variable [105, 106].

## Discussion

This research is highly significant in several ways. First, if successful, reducing the material and psychological burden of financial toxicity will improve the quality of cancer care. Second, the intervention can easily be adapted to other cancers, where expensive treatments are emerging. Third, this research will provide empirical data regarding the mechanisms through which treatment cost discussions and other aspects of clinical communication improve patient outcomes related to financial toxicity. Finally, this research directly addresses disparities in cancer care by improving communication quality for patient groups suffering the disproportionate burden of disparities in the financial consequences of cancer care.

This study is not without potential limitations. One of these is the focus on oncologists, rather than or in addition to other providers, such as nurses and social workers, who are instrumental to helping patients navigate the financial issues related to treatment and survivorship. However, we focus on oncologists because they, with their patients, make the final treatment decisions that have the greatest influence on financial consequences. Also, patients consider physicians to be their primary and preferred source of information. Second, it is possible that some patients may not be comfortable using iPads. However, our feasibility testing and the prevalence of smartphones and app usage in this population lead us to believe this will be of limited concern [79, 89, 107]. The app-based nature of this intervention enhances its scalability and dissemination. Third, given the multiple patient follow-ups, attrition is a concern. We will employ several strategies to keep attrition to a minimum [48, 53, 56, 96], First, we will compensate patients up to $150 in gift cards, which they will receive in increments. Patients will receive $20 after each video-recorded interaction and after the first three follow-up surveys. As a further incentive, they will receive $50 after they complete the final follow-up survey. Second, patients will receive email and text reminders 2 days before each survey, depending on their preference for contact. Third, because of concern about patient burden, we will keep measures to a minimum. Last, we will monitor for attrition and adjust our methods if needed. These recruitment and retention procedures have been quite successful in our prior studies [53, 86, 96].

### Dissemination

Our research team is well-positioned to disseminate our preliminary and final findings. We have ongoing collaborations with several community-based cancer education and advocacy groups through KCI’s Office of Cancer Health Equity and Community Engagement (OCHECE). These groups, called Cancer Action Councils (CAC), are located throughout Metro Detroit and CAC members served as key informants at the earliest stages of the DISCO App’s development. Dr. Hamel (first author/PI) is also a member of the Michigan Cancer Consortium (MCC), a statewide, broad-based partnership of public and private organizations that provides a forum for collaboration to reduce the burden of cancer among the residents of Michigan. Dr. Hamel is also an active member of several professional organizations including ASCO, the American Academy of Communication in Healthcare/European Academy of Communication in Healthcare (AACH/EACH), and the Society of Behavioral Medicine (SBM).

If we demonstrate the DISCO App’s effectiveness, we can disseminate our findings to community-, state-, and nationally focused organizations, in addition to presenting our interim and final findings to academic conferences and high-impact scientific journals. Furthermore, the DISCO App is a product, and if found to be effective, we are in a position to disseminate the DISCO App in KCI’s out-patient clinics across the state of Michigan, thus contributing an evidence-based tool to reduce financial toxicity in diverse populations.

### Responsibility of the coordinating center

All research activities will occur at KCI/WSU, which will also serve as the study’s coordinating center. Dr. Hamel (PI) is based at KCI/WSU, and she will oversee all scientific and administrative aspects of the recruitment of physicians and patients, implementation of the intervention, data collection and analysis, and preparation of reports and manuscripts.

### Data monitoring

A data monitoring committee (DMC) comprising the principal investigator and co-investigators will monitor all self-report, video, observational, and medical record data throughout the duration of the study. The DMC will operate independently from the funder. The biostatistician on this study will conduct interim audit analyses quarterly. Following the WSU IRB rules and definitions, adverse and expected events will be reported to the WSU IRB. The principal investigator will be responsible for all aspects of the trial and will make the final decision when to end the trial.

All data files will be stored on a network server at the KCI/WSU in the Department of Oncology. Only the WSU staff listed in the application will have access to the files and at no time will data files be shared with collaborators outside the institution. The KCI/WSU network server utilizes hardware-based encryption at the level of the hard drives. Approved domain users are granted project-specific permission on the server folders.

The server is backed up to an off-site software-encrypted disk-based backup solution. Dr. Hamel’s computer and network server are protected under the same CISCO firewall. The recordings will be kept in a locked file cabinet in KCI’s Behavioral and Field Research Core’s editing suite (which is also locked whenever unoccupied). Patient medical record numbers will be assigned a study ID number in a master key, and study IDs will be used on all research documents. Only the principal investigator, co-investigators, and data manager will have access to the master key, which will be locked in password-protected computers as described. We assure that any publications and presentations of the data will not allow for the identification of patients, hospitals, or physicians.

### Trial status

Protocol # 2020-117 was approved on October 21, 2020, by Karmanos Cancer Institute’s Protocol Review and Monitoring Committee and approved on December 17, 2020, by Wayne State University’s Institutional Review Board (# IRB-20-10-2836-B3).

Recruitment began on March 10, 2021, and will continue until approximately September 30, 2023.

## Data Availability

Not applicable

## Abbreviations

AACH/EACH: American Academy of Communication in Healthcare/European Academy of Communication in Healthcare
ASCO: American Society of Clinical Oncology
CAC: Cancer Action Councils
DMC: Data monitoring committee
DISCO App: DIScussions of COst App
GEE: Generalized estimating equations
ICC: Intra-cluster correlation
KCI: Karmanos Cancer Institute
MLM: Multi-linear models
OCHECE: Office of Cancer Health Equity and Community Engagement
QPLs: Question prompt lists
RCT: Randomized controlled trial
RAs: Research assistants
SW/FN: Social worker/financial navigation
SBM: Society of Behavioral Medicine
WSU: Wayne State University
